# Ecosystem degradation and the spread of Covid-19

**DOI:** 10.1007/s10661-023-11403-6

**Published:** 2023-06-13

**Authors:** Chiara Castelli, Marta Castellini, Nicola Comincioli, Maria Laura Parisi, Nicola Pontarollo, Sergio Vergalli

**Affiliations:** 1 The Vienna Institute for International Economic Studies, Vienna, Austria; 2 Department of Economics and Management “Marco Fanno”, University of Padua, Padua, Italy; 3 Fondazione Eni Enrico Mattei, Milan, Italy; 4 Department of Economics and Management, University of Brescia, Brescia, Italy

**Keywords:** Early spread of SARS-CoV-2, Human appropriation of net primary production (HANPP), World regions, Regression analysis, Bayesian estimation, Spatial random effects, C11, C21, R10

## Abstract

The linkages between the emergence of zoonotic diseases and ecosystem degradation have been widely acknowledged by the scientific community and policy makers. In this paper we investigate the relationship between human overexploitation of natural resources, represented by the Human Appropriation of Net Primary Production Index (HANPP) and the spread of Covid-19 cases during the first pandemic wave in 730 regions of 63 countries worldwide. Using a Bayesian estimation technique, we highlight the significant role of HANPP as a driver of Covid-19 diffusion, besides confirming the well-known impact of population size and the effects of other socio-economic variables. We believe that these findings could be relevant for policy makers in their effort towards a more sustainable intensive agriculture and responsible urbanisation.

## Introduction

As the world's population grows, the conflict between ecosystem conservation and the demand for food becomes more difficult to manage every day (Crist et al., 2017; Fischer et al., 2017). Numerous policies have attempted to mitigate this well-known problem in the long-standing dilemma of allocation between protected areas and agricultural land,^1^ promoting the sustainable intensification of agriculture.^2^ However, Covid-19 outbreak has emphasized the negative side effects of decades of human overexploitation of land and natural resources (see e.g. Foley et al., 2005, Altieri & Nicholls, 2020 and McNeely, 2021) and of energy-intensive agriculture (Agnoletti et al., 2020).

According to the Kenyon (2020) review, the rate of emergence of zoonoses has increased over the last 40 years.^3^ The author suggests approaching zoonoses through an eco-social conceptual framework of health and disease. According to this view, two different perspectives should be considered when discussing the determinants of the spread of viruses, namely the close interaction of humans with wildlife and related consumption—individual dimension—and the anthropogenic environmental degradation—eco-social dimension. The author argues that, if an eco-social conceptual framework had been followed, decision-makers would have had to take steps to reduce human interaction with natural habitats in order to preserve ecological systems. This would also have prevented or slowed the spread of SARS-CoV-2.

McNeely (2021) discusses a similar approach, comparing the bubonic plague pandemic of the mid-fourteenth century with the recent Covid-19 one. In both cases, awareness of the possible environmental causes of these events has been raised and compensatory measures have been considered, involving a rethinking of society towards a more sustainable and resilient paradigm. While deepening the central role of biodiversity loss and the disruption of natural ecosystems in the emergence of the Covid-19 pandemic, the author describes past events, such as the Ebola case of 1976 in West Africa, as an example of how deforestation brought wild species into contact with humans and spread infectious diseases. It is not by chance that the service provided by the ecosystems^4^ in regulating the emergence and spread of diseases, was recognized as the central core of the 2005 Millennium Assessment classification of ecosystem services underpinning human wellbeing (Everard et al., 2020). The EcoHealth Alliance (2019) reports that land use changes account for 31% of the primary drivers of infectious diseases that have originated in wildlife since 1940. On this side, the recent scientific literature agrees that land use changes increase the risk of zoonotic disease emergence (see, among others, Brearley et al., 2013; Gottdenker et al., 2014; Gibb et al., 2020; Myers et al., 2013 and Brancalion et al., 2020). Gibb et al. (2020), for example, carry out an analysis of 6,801 ecological assemblages and 376 host species worldwide based on the PREDICTS database^5^ developed by Hudson et al. (2017), finding that the conversion of natural environments to agricultural sites or urban areas has systematic effects on local zoonotic host communities. The review by Gottdenker et al. (2014) lists specific types of land use change that are associated with disease spread, namely deforestation, forest and habitat fragmentation, agricultural development, irrigation, urbanisation and suburbanisation. The mechanisms by which these interventions are linked to the transmission of infectious diseases include changes in the spatial distribution of hosts and/or vectors, socio-economic factors and environmental contamination, although there is still considerable uncertainty about the magnitude.

The importance of ecosystem restoration has also been formalised with the establishment of the United Nations Decade of Ecosystem Restoration 2021–2030. Robinson et al. (2022) discuss the importance of restoring ecosystems as part of the path out of Covid-19 that can ensure health and socio-economic stability, although such integration in the responses to disease is poorly represented at the time of the analysis. For this reason, they call for improvements in policy developments towards this direction, combined with evidence-based tools to guide policymakers.

In this paper, following the perspective of Kenyon (2020), we implement an empirical analysis aimed at identifying what he defines as “causal factors underpinning the emergence of zoonoses such as SARS-CoV-2”. Specifically, we evaluate the impact of human overexploitation of natural resources as a potential driver of the 2019 pandemic outbreak using a Bayesian estimation technique based on a cross-section of 730 regions in 63 countries worldwide. While controlling for several socio-economic, health and climate related covariates, we concentrate on the role of the Human Appropriation of Net Primary Production (HANPP), an index introduced by Imhoff et al. (2004) to measure land use and over exploitation, as a potential ley explanatory variable of the early Covid-19 outbreak. The HANPP captures “*human alterations of photosynthetic production in ecosystems and the harvest of products of photosynthesis*” showing “*the aggregate impact of land use on biomass available each year in ecosystems*”, as Haberl et al. (2007) explain.

Results obtained in this analysis confirm that land overexploitation has a significant role in the early spread of the Covid-19 pandemic. The findings, robust to a range of alternative model specifications and estimation techniques, provide useful information for the calibration of policies against ecosystems overexploitation and the zoonosis prevention as well. Despite the hypothesis that a reduction in ecosystem services may ease the spread of infectious diseases has already been proposed in the literature (see, e.g., Morand & Lajaunie, 2018), to the best of our knowledge, this is the first quantitative study using the HANPP as an explanatory variable while also discussing implications related to the Covid-19 pandemic. It also contributes to the literature on geographical patterns of connectedness and embeddedness that help explaining where the pandemic hit the most (see e.g. Amdaoud et al., 2021, for the EU and Sun et al., 2020, for the US), by including in our study regions all over the world and a spatial econometric modelling approach.

The remainder of the paper is organised as follows. In the second section we describe the context and we provide a brief literature review. In section three we present the model and the data. Results are reported in section four and, finally, section five concludes and draws some policy implications.

## Context and literature review

### Ecosystem degradation and the link with human infections

There is a wide consensus about the central role of biodiversity loss as a key driver of emerging infectious diseases (see Everard et al., 2020; Kenyon, 2020; McNeely, 2021 and Olivero et al., 2017, among others). The growing interaction between humans and wildlife, due to the continuous reduction of intact natural habitats, is causing an increase of human diseases of animal origin, also known as zoonoses. According to Woolhouse (2002), the emergence of most pathogens is commonly associated with ecological change and three-quarters of emerging human pathogens are zoonotic.

Intense human activities have undermined the natural evolution of ecosystem services such as the availability of fresh water provision, essential for hygiene, to prevent human-to-human transmission and for treating resultant infections (Everard et al., 2020), as well as natural barriers, accelerating the occurrence of natural disasters and floods. Moreover, human appropriation of natural resources negatively affects the conversion of solar energy into organic carbon compounds, a process performed by water bodies algae (seaweed, algae diatoms) as well as by all the terrestrial plants during the photosynthesis process (EC, 2019). As stated by Haberl et al. (2007), land use transforms Earth’s terrestrial surface, leading to changes in biogeochemical cycles and in the ability of ecosystems to deliver services critical to human wellbeing. The output of this global conversion is referred to as Net Primary Production (NPP).^6^ All organisms, e.g., all species of animals including humans, bacteria, fungi, depend directly and indirectly on the primary production of plants as an essential foundation of their livelihood (EC, 2019). The disproportionate usage of NPP will be the focus of our extended analysis. Specifically, the key variable of this study is represented by the Human Appropriation of Net Primary Production (HANPP) estimated by Imhoff et al. (2004), expressed as grams of carbon per grid cell of 0.25 decimal degrees, approximately 28 km on a side at the equator. For each of the 730 regions in the sample, the average HANPP is calculated (and the logs are taken).

HANPP values are given by the sum of loss of potential NPP due to land use change and the NPP harvested by humans, both measured as annual carbon flows. The former, in turn, consists of the difference between the Net Primary Production “Supply” and “Demand”. Following the definitions in Haberl et al. (2007), the first can be also defined as the natural capacity of primary biomass production of “undisturbed” terrestrial ecosystems (i.e. under current environmental conditions), while the latter is the quantity that remains after anthropogenic land conversion and biomass harvesting of all types (i.e. not only agricultural crops). A high value of HANPP indicates a high level of ecosystem degradation.

The complexity of this index emerges from the difficulty to update it with more recent data (land use data, for example, at national level are not consistently mapped, see Seeber et al., 2022 for a discussion), and from the fact that its values include the impact, on the amount of global carbon flows assimilated by vegetation, of human-induced land conversions, such as land cover change, land use change, and soil degradation (Haberl et al., 2007).^7^ However, in spite of these limitations Krausmann et al. (2012) managed to calculate the HANPP up to year 2000, but for only six countries based on a very long time series of available observations. Kastner et al. (2021) made another attempt, managing to compute a version of the HANPP index at a resolution of five arcminutes between 1910 and 2010 for 9 points in time. However, their index used a methodology that differs from Imhoff et al. (2004), focusing essentially on land use, rather than on a set of variables proxying human exploitation of the environment. The difference between the two ways in which the HANPP indexes are calculated is demonstrated by the correlation equal to 0.003. In our work we consider that HANPP based only on land use does not provide useful information for the scope of our study and therefore we rely on the original version by Imhoff et al. (2004).^8^

**Fig. 1:**
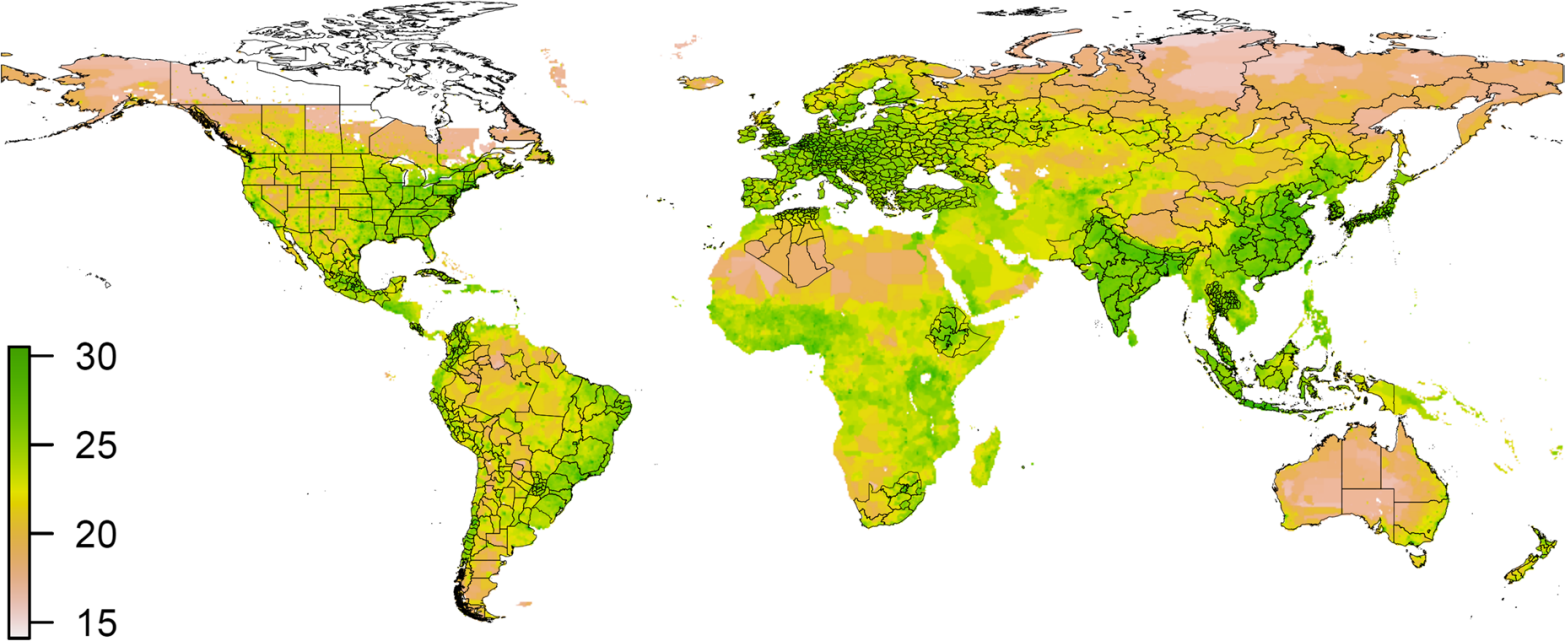
Global Map of the log of HANPP in 2000 (log of grams of carbon per grid cell). Note: regions in the map represent the sample used in the study. Source: own elaboration based on Imhoff et al. (2004)

Figure 1 represents a global plot of grid-based HANPP values for the reference year 2000. In terms of natural resources overexploitation, the most critical areas (green colour) belong to intense agricultural and industrialized regions, also characterized by high population density (e.g. Eastern and Southern Asia, India, Europe and North America). On the other hand, areas of scarce human activity, such as the African Saharan region, Mongolia and Siberia (Russia) register low levels of human appropriation of biomass production (pink colour).

Although the relationship between the degradation of ecosystems and zoonoses has been widely discussed and empirical evidence is abundant (see Keesing et al., 2006, 2010; Ostfeld & Keesing, 2017; Suzán et al., 2009 among others), even related to the Covid-19 (e.g. Mishra et al., 2021; Wu, 2021), research close to our goal is still lacking. Indeed, rather than identifying if pathogen emergence is related to degradation of ecosystems, we want to check if the latter can facilitate the spread of the Covid-19 pandemic in its initial stage, using regional data worldwide. To the best of our knowledge, a similar approach has been adopted only by Fernández et al. (2021), who have studied the same topic at country level (on a sample of 160 countries), finding a significant relationship between the loss of biodiversity and Covid-19 infection spread and mortality. On the other hand, Solimini et al. (2021), from which we take part of our data (see next section), demonstrates a correlation between airborne particulate concentration and Covid-19 spread using worldwide regional data. According to this literature, if air pollution continues over time, degradation will grow, damaging ecosystems until a breaking point from which it may be difficult to recover (the so-called “tipping point” of De Zeeuw & Li, 2016).

## Other environmental and human drivers of pathogens diffusion

In addition to environmental degradation, the spread of infectious diseases among humans may also be linked to the demographic and socioeconomic characteristics of local communities (Ying et al., 2022). With regard to the former, many studies have considered the role of population size (Stier et al., 2020, 2021) and density (Hu et al., 2013; Kraemer et al., 2015; Liu, 2020, among others). According to the urban scaling theory (Bettencourt, 2013), population size can act as a multiplier for many socioeconomic outcomes such as crime rates, the number of patent applications, as well as the rapid spread of infections among individuals. Similar to population size, another important urban characteristic that favours frequent human contact, which may imply a faster transmission of diseases, is population density. However, studies considering its specific role on the spread of human pathogens seem to find different results. For example, Hamidi et al. (2020) justify a non-significant relationship between population density and Covid-19 infection rates arguing that adherence to social distancing policies is greater in denser areas, where there is also better quality health care. Non-significant results are also found in Boterman (2020) when controlling for socio-economic factors. In line with this view, which sees population density as a proxy for higher civic engagement and better-quality infrastructure, Liu (2020) finds a negative correlation with the spread of Covid-19 in the early stages of the epidemic in China. Another relevant demographic characteristic that should be considered as a potential driver of pathogens diffusion is age. In particular, higher rates of mobility are expected among younger age groups than among the elderly ones due to schooling, work and social life, which imply more frequent human contact. This, in turn, would make the young population an effective vehicle for the spread and transmission of Covid-19 (Monod et al., 2021), albeit with less severe consequences in terms of illness and death risk compared to older age groups (Coker, et al., 2020; Iacus et al., 2020; Zheng et al., 2021). Moreover, as infections can be related to each other and/or to other clinical conditions, for the choice of our covariates we follow the specific Covid-19 literature, which highlights diabetes as a significant predictor of the virus mortality (Corona et al., 2021).

In terms of socio-economic aspects, the standard of living, the quality of health-related infrastructure and the economic development of a given area are factors related to the broader concept of human development, which has been identified in the literature as a significant driver of population health outcomes (see Solimini et al., 2021 and Chen et al., 2021, among others). According to Solimini et al. (2021), economic output per capita can be seen as a proxy for both health infrastructure and population health status (e.g. in terms of life expectancy and infant mortality), as well as economic development (Chen et al., 2021). Regarding the specific Covid-19 literature, Sigler et al. (2021) find the Human Development Index (HDI) to be a strong predictor of its diffusion, especially in the early phase of the pandemic. Further support for this finding is provided by Khazaei et al. (2020) and Zhou and Puthenkalam (2022).

Finally, environmental conditions seem to play a relevant role in the transmission of respiratory diseases, especially when considering meteorological factors and air pollution. With regard to the former, temperature appears to be a much-cited factor in reducing the spread of Covid-19 (for a review, see the work of Han et al., 2022), although this negative effect needs to be interpreted with caution in view of the significant spatial heterogeneity shown in several studies.^9^

Another important meteorological factor that can facilitate the transmission of the virus is humidity, which contributes to its viability and persistence on inanimate objects (Sarkodie & Owusu, 2020; Zarei et al., 2021). In particular, evidence shows that Covid-19 can spread more rapidly with humidity (Park et al., 2020; Wu et al., 2021) due to an increase of droplets lifetime (Chen et al., 2021).

Air pollution is another critical element that can affect both the spread and deadliness of respiratory viral infections, as highlighted in the SARS-CoV-2 literature (Han et al., 2022; Solimini et al., 2021). In particular, specific attention has been paid to the potential effects of particulate matter (PM 10 and PM 2.5) on the initial spread of the epidemic, as this pollutant can (i) facilitate the entry of viruses into the human body and (ii) hinder the immune system response to these viruses (Solimini et al., 2021).^10^ Furthermore, in a study conducted by Zhu et al. (2020) for China, short-term exposure to NO2 or O3 had a greater effect than PM 2.5 or PM 10 in increasing the number of Covid-19 confirmed cases, thus highlighting a certain degree of heterogeneity even within the same group of air pollutants (as well found in the global study of Solimini et al., 2021).

It is easy to see that these studies on the transmission and deadliness of human pathogens resulting from the effects of air pollution, as well as those coming from the loss of biodiversity and ecosystem richness discussed in the previous section, share a common denominator: the overexploitation of natural resources by human activities.

## Methodology and data

**Fig. 2:**
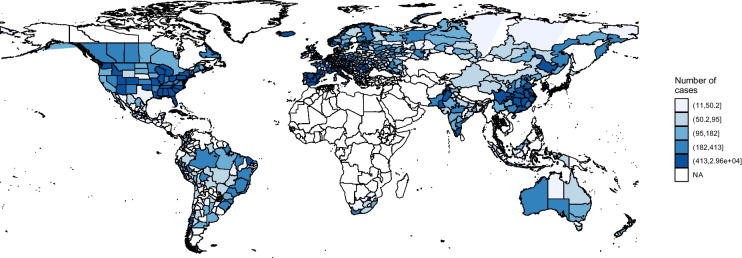
Covid-19 cumulative cases registered in the following 14 days from the date when cases are > 10. Note: regions in the map represent the sample used in the study. Source: own elaboration based on Solimini et al. (2021)

In order to investigate the potential effect of the HANPP Index on the spread of Covid-19, as customary in the literature, we rely on a negative binomial mixed model. More specifically, as a dependent variable we use the number of Covid-19 cumulative cases registered in the following 14 days from the date when > 10 cumulative cases are reported for 730 regions in 63 countries (Solimini et al., 2021). This leads to include information up to May 30, 2020. Figure 2 shows the reported cases. The highest incidence of the early spread of Covid-19 is observed in Europe, in the Eastern part of the U.S., in China and the coastal regions of Brazil (areas of darker blue colour), while Mongolia, Siberia (Russia), Central American countries and Latin American countries bordering the Pacific Sea (light blue colour) showed the lowest incidence.

The estimates are performed using a Bayesian hierarchical approach via Integrated Nested Laplace Approximation (INLA), see Rue et al. (2009). This is a computationally efficient alternative to Markov chain Monte Carlo (MCMC) methods (Rue & Held, 2005).

We employ a set of different Bayesian models starting from the benchmark, including only the covariates, adding step-by-step national, continental or spatial random effects.^11^ As a further robustness check we combine spatial random effects to country or continent fixed effects. Our model has the following form:

1 $$\begin{array}{c}{CovidCases}_{i}\sim {NB}_{i}\left({\mu }_{i},\theta \right)\\ \mathrm{log}\left({\mu }_{i}\right)=\alpha +\beta {HANPP}_{i}+{\delta }^{^{\prime}}{X}_{i}+{u}_{i}+{\varepsilon }_{i}\end{array}$$

where θ is the overdispersion parameter^12^ of a Negative Binomial distribution and *μ* is the region-specific expectation conditional on the value of the covariates. *HANPP* is the Human Appropriation of Net Primary Production in region *i* and β is the associated parameter; ***X*** is a vector of control variables that adjusts for the potential confounding effects and includes the (log of) total population as the offset with ***δ*** the associated vector of parameters. Parameter ${u}_{i}$ represents the random effect corresponding to region $i$ (see footnote 4) and ${\varepsilon }_{i}$ is a normally-distributed error term.

The covariates, coherently with what reported in Sect. 2, include socio-economic, demographic and environmental factors. Table 1 presents the source, the scale (regional or national) and the definition of each variable, in addition to the descriptive statistics. In Table 3 (Appendix A) we report the covariance matrix among the regressors.

**Table 1:** Description of the variables

Variable	Source	Scale	Definition	Min	Mean	Std.Dev	Max
Cumulative Cases	Solimini et al. (2021) based on various sources	Regional	cumulative number of cases in the 14 days following the date when > 10 cumulative cases were reported	11	339	1266.7	29,631
Demographic Variables							
Log Total Population	Solimini et al. (2021) based on Gridded Population of the World (version 4.11, distributed by Socioeconomic Data and Applications Center)	Regional	total population	9.283	14.467	1.392	18.657
Population Age 65 +	Solimini et al. (2021) based on Gridded Population of the World (version 4.11, distributed by Socioeconomic Data and Applications Center)	Regional	Share of population of age 65 + (%)	0.769	10.881	5.266	25.105
Proportion Males	Solimini et al. (2021) based on Gridded Population of the World (version 4.11, distributed by Socioeconomic Data and Applications Center)	Regional	Share of males in the population (%)	45.049	49.39	1.604	53.23
Socio-Economic Variables							
Prevalence Diabetes	Solimini et al. (2021) based on World Bank	National	prevalence of diabetes (%)	3.2	7.516	2.701	19.9
Log Num. of Test	Solimini et al. (2021) based on World Bank	National	cumulative number of test at the outcome date	5.421	11.396	1.72	15.468
Stringency Index	Solimini et al. (2021) based on ourworldindata.org/	National	stringency index at the outcome date	2.78	77.53	14.894	100
log Income Index	Smits and Permanyer (2019)	Regional	GDP per capita in thousands of USD (2011 PPP)	8.101	10.032	0.662	12.096
log Schooling Level	Smits and Permanyer (2019)	Regional	Mean years schooling population aged 25 +	3.1	10.811	2.119	14.47
log Population Weighted Dens	Global Human Settlement Layer	Regional	Population weighted density passed on gridded population at 1 square km in 2015	-1.107	2.537	0.871	5.523
Environmental Variables							
Humidity	Solimini et al. (2021) based on Copernicus Climate Change Service (C3S)	Regional	max relative humidity in the 30 days before outcome date	29.486	69.03	10.609	90.5
Temperature	Solimini et al. (2021) based on Copernicus Climate Change Service (C3S)	Regional	mean temperature in the 30 days before outcome date	-13.464	15.667	8.229	31.898
PM 2.5 Concentration	Solimini et al. (2021) based on Copernicus Atmosphere Monitoring Service (CAMS)	Regional	Mean PM 2.5 concentration for the period 2014–2018 based on daily data for the cell spatially closest cell to the geographic coordinates of the location with higher population size within each administrative unit	2.387	23.922	33.625	163.46
log HANPP	Imhoff et al. (2004)	Regional	Net primary production ‘demand’ minus ‘supply’—log of grams of carbon	18.522	25.17	1.69	30.7

As far as the demographic variables are concerned, in addition to the total population in the region, as the offset, we include the proportion of male population, as well as the proportion of the elderly population (i.e. those aged 65 and over) in order to take into account such highly vulnerable age group exposed to infections, especially when affected by multiple pathologies (Dadras et al., 2022). In the vector of socio-economic variables, we account for income per capita and educational attainment, measured as average years of schooling in the population aged 25 and older that come from Smits and Permanyer (2019). Their inclusion is justified by their importance in explaining Covid-19 cases and deaths. Examples are Hawkins et al. (2020) for the U.S., Cifuentes et al. (2021) for Colombia, Mateo-Urdiales et al. (2020) for Italy and Meurisse et al. (2022) for Belgium. Population Weighted Density (PWD), based on gridded population at 1 square km in 2015, is not only recognized as a better measure of density at which the population lives (Craig, 1984), but it is currently a good predictor of the spread of Covid-19 pandemic (Baser, 2021; Wong & Li, 2020). Additionally, in the same vector, we have a set of health variables compiled by Solimini et al. (2021) from various sources, consisting in the national prevalence of diabetes,^13^ the cumulative number of tests at outcome date and stringency index at outcome date, i.e. a composite measure based on nine response indicators including school and workplace closures and travel bans, rescaled to a value from 0 to 100 (100 = stricter response), as in Hale et al. (2021).^14^ Finally, following Chen (2020) and Ma et al. (2021) in the vector of environmental factors there are the maximum relative humidity and the mean temperature in the 30 days before the outcome date, and the mean PM 2.5 concentrations for the period 2014–2018 as in Coker et al. (2020).

To confirm the reliability of the results, we have executed two robustness checks, based on the observation of the cumulative number of Covid-19 cases starting from the 30th or 45th day after the tenth case is observed. These further results are provided in Appendix B.

## Results

Six different specifications of model (1) have been estimated in the empirical part of this study: (i) baseline fixed effects model, (ii) country random effects, (iii) continent with nested country random effects, (iv) spatial random effects, (v): model (iv) plus country fixed effects, and (vi): model (v) plus continent fixed effects. Bayesian estimates of the coefficients are shown in Table 2. All variables, including the intercept, have predictive power on Covid-19 early diffusion, at least in one model specification. In addition, the analysis of the Deviance Information Criterion (DIC) reveals the preference for the model with random effects accounting for the geographical locations, the best of which is model specification (ii).

**Table 2:** Estimation results

Model Specification	Baseline			Country R.E			Continent +		
							**Nested Country R.E**		
	**(i)**			**(ii)**			**(iii)**		
Variable	**Mean**	**0.025 q**	**0.975 q**	**Mean**	**0.025 q**	**0.975 q**	**Mean**	**0.025 q**	**0.975 q**
Intercept	**-5.3550**	**-7.1416**	**-3.5076**	-2.7208	-5.3868	0.0237	**-2.7700**	**-5.4307**	**-0.0273**
Demographic Variables									
log Total Population	**0.2421**	**0.1739**	**0.3097**	**0.3049**	**0.2285**	**0.3809**	**0.3040**	**0.2274**	**0.3799**
Population Age 65 +	0.0187	-0.0016	0.0390	-0.0211	-0.0504	0.0079	-0.0208	-0.0501	0.0082
Proportion Males	**-0.0502**	**-0.0748**	**-0.0269**	**-0.0862**	**-0.1140**	**-0.0609**	**-0.0857**	**-0.1134**	**-0.0605**
Socio-Economic Variables									
Prevalence Diabetes	**0.0378**	**0.0092**	**0.0670**	-0.0306	-0.1128	0.0488	-0.0294	-0.1116	0.0503
log Number of Test	-0.0097	-0.0285	0.0087	0.0084	-0.0174	0.034	0.0076	-0.0182	0.0332
Stringency Index	**0.0110**	**0.0063**	**0.0154**	0.0034	-0.0037	0.0103	0.0033	-0.0038	0.0102
log Income Index	**0.8249**	**0.6701**	**0.9781**	**0.5114**	**0.2841**	**0.7361**	**0.5140**	**0.2865**	**0.7391**
log Schooling Level	**-0.0812**	**-0.1340**	**-0.0282**	**0.0952**	**0.0109**	**0.1799**	**0.0934**	**0.0091**	**0.1781**
log Population Weighted Density	0.0232	-0.0734	0.1211	0.0604	-0.0521	0.1734	0.0605	-0.0520	0.1736
Environmental Variables									
Temperature	**-0.0421**	**-0.0559**	**-0.0283**	-0.0003	-0.0172	0.0164	0.0001	-0.0168	0.0168
Humidity	**0.0118**	**0.0048**	**0.0187**	**0.0141**	**0.0061**	**0.0220**	**0.0142**	**0.0063**	**0.0221**
PM 2.5 Concentration	**0.0045**	**0.0017**	**0.0074**	**0.0062**	**0.0030**	**0.0095**	**0.0062**	**0.0030**	**0.0095**
log HANPP	**0.0356**	**0.0077**	**0.0604**	**0.0346**	**0.0077**	**0.0589**	**0.0348**	**0.0079**	**0.0591**
Country random effects				1.7389	1.0454	2.7089	1.7282	1.0373	2.6904
DIC	9420.3			9098.7			9099.2		

Model Specification	Spatial R.E			Spatial R.E. + Country F.E			Spatial R.E. + Continent F.E. + Country F.E		
	(iv)			(v)			(vi)		
Variable	**Mean**	**0.025 q**	**0.975 q**	**Mean**	**0.025 q**	**0.975 q**	**Mean**	**0.025 q**	**0.975 q**
Intercept	**-5.3578**	**-7.1206**	**-3.5368**	-0.9642	-22.4811	20.5364	-1.1392	-31.6915	29.3886
Demographic Variables									
log Total Population	**0.2421**	**0.1749**	**0.3088**	**0.3093**	**0.2265**	**0.3919**	**0.3101**	**0.2273**	**0.3926**
Population Age 65 +	0.0187	-0.0014	0.0388	**-0.0413**	**-0.0753**	**-0.0075**	**-0.0406**	**-0.0746**	**-0.0068**
Proportion Males	**-0.0502**	**-0.0745**	**-0.0272**	**-0.0915**	**-0.1217**	**-0.0646**	**-0.0919**	**-0.1221**	**-0.0650**
Socio-Economic Variables									
Prevalence Diabetes	**0.0378**	**0.0096**	**0.0666**	-0.1041	-2.4747	2.2647	-0.1256	-2.5265	2.2734
log Number of Test	-0.0097	-0.0283	0.0084	0.0063	-0.022	0.0344	0.0062	-0.0221	0.0343
Stringency Index	**0.011**	**0.0064**	**0.0154**	0.0051	-0.0038	0.0136	0.0051	-0.0038	0.0136
log Income Index	**0.8249**	**0.6722**	**0.976**	**0.4029**	**0.1431**	**0.6599**	**0.3865**	**0.1254**	**0.6449**
log Schooling Level	**-0.0812**	**-0.1333**	**-0.029**	0.0771	-0.0247	0.1796	0.0792	-0.0227	0.1817
log Population Weighted Density	0.0232	-0.0721	0.1198	0.1100	-0.0134	0.2337	0.1105	-0.0128	0.2341
Environmental Variables									
Temperature	**-0.0421**	**-0.0557**	**-0.0285**	0.0151	-0.003	0.033	0.0151	-0.0030	0.0329
Humidity	**0.0118**	**0.0049**	**0.0186**	**0.0137**	**0.0052**	**0.0221**	**0.0138**	**0.0053**	**0.0222**
PM 2.5 Concentration	**0.0045**	**0.0018**	**0.0074**	**0.0063**	**0.0028**	**0.0100**	**0.0063**	**0.0028**	**0.0100**
log HANPP	**0.0356**	**0.0081**	**0.0601**	**0.0387**	**0.0100**	**0.0645**	**0.0387**	**0.0100**	**0.0644**
Country random effects									
DIC	9418.4			9104.2			9104.4		

bold indicates coefficients statistically different from zero at 5% level

Consistently with the previous literature (Stier et al., 2020, 2021), population size has a significant positive estimated impact on the early diffusion of Covid-19 in all specifications, while the share of male population has a significant negative effect, as in Coker et al. (2020). Unlike other studies (see, e.g., Yanez et al., 2020), the share of population aged 65 and over is never significant either in the main results, with the only exception of model (v) and (vi), or in the robustness checks.

This result can be reasonably explained by the tendency of the elderly to have lower mobility, not influencing Covid-19 spread, at least in the very early stage of diffusion (with fewer exceptions, e.g. the North of Italy, where the elderly suffered immediately from the diffusion and mortality risk of the virus, as documented in Grasselli et al., 2020). However, as the elderly tend to congregate often for recreational activities, in the second phase of the pandemic this population group was fatally affected almost everywhere (Coker, et al., 2020; Iacus et al., 2020; Zheng et al., 2021).

Results appear heterogeneous in terms of socio-economic variables. Income per capita, proxying the economic development of a region, has always significant and positive effects on new cases of Covid-19, confirming previous results (see, e.g., Chen et al., 2021). This result highlights a potential link between the circulation of Covid-19 and human development, conceived as an indicator of frequent human contacts and close social ties among people. The effect of schooling, positive and significant in our best models (ii) and (iii), i.e. those with lowest DIC, can be read following the same reasoning. These results are in line with Sigler et al. (2021) who show that, in the early stage of the pandemic, human development index (HDI) is the strongest predictor of new cases, pointing to a hierarchical diffusion from more developed countries to less developed ones. The positive correlation between HDI and the spread of Covid-19 is well documented in literature, both at the global level (Khazaei et al., 2020) and with a focus on high-income countries (Zhou & Puthenkalam, 2022). Finally, a possible concern regarding the interdependence between variables representing socio-economic regional factors and HANPP is generally excluded due to i) the low correlation reported in Table 3 in Appendix A, ii) the evidence that results hold even when HANPP is removed from the specification, and iii) the fact that HANPP is computed for year 2000, while other covariates at year 2020. This last point allows us also to limit the concerns regarding reverse causality.

The results for the socio-economic variables available at national level, namely the prevalence of diabetes, the stringency index and the number of cumulative tests, are either weakly or not significant in all our specifications. This highlights the need for more appropriate measures to test the specific role of both regulatory and preventive health security measures taken by policymakers over time. Specifically, a weak positive relationship is observed for the prevalence of diabetes and the stringency index, whose coefficients are significant only in two cases (i and iv). However, the number of tests is never significant (testing and contact tracing methods were not so developed yet at the observed time).

Interestingly, the coefficient of PWD is also found to be insignificant although this lack of effect may be an important result that can corroborate other studies (as discussed below), rather than highlighting a misspecification measurement. Indeed, several scholars such as Hamidi et al. (2020) and Boterman (2020) account for both population density and concentration, as the PWD does, and find non-significant results. The first authors argue that adherence to social distancing policies and practices is greater in denser areas, where better quality of health care can be also found, and this is why they find no relation with infection rates and a negative relationship with the mortality rate. Boterman (2020), on the other hand, finds that, when controlling for socio-economic factors, density loses statistical significance. Nonetheless, the debate on the effect of population density on the Covid-19 spread should be still considered an open issue, which seems to depend on several factors, such as the way in which density is measured, the specific case-study and the phase of the pandemic (see Ying et al., 2022, among others).

As far as environmental factors, humidity, coherently with the literature reported in Sect. 2, has always a significant positive effect on our dependent variable, while temperature appears to produce a weak negative effect (observed in two model specifications, i and iv). These results are in line with the model proposed by Chen (2020), according to which droplets’ lifetime is strongly influenced by humidity, while temperature appears to be less relevant.

Finally, our results confirm the evidence by Solimini et al. (2021) about the positive and significant effect of particulate matter (PM 2.5) on the diffusion of Covid-19, even when additional variables are included in the specification. For example, in the most effective model specification (ii), we find that a unit increase of PM 2.5 (μg/m^3^) is correlated with a 0.7% increase in the dependent variable. Such a result further confirms what was found in the literature by Coker et al. (2020) at local level and Fernández et al. (2021) at global scale, among others.

**Fig. 3:**
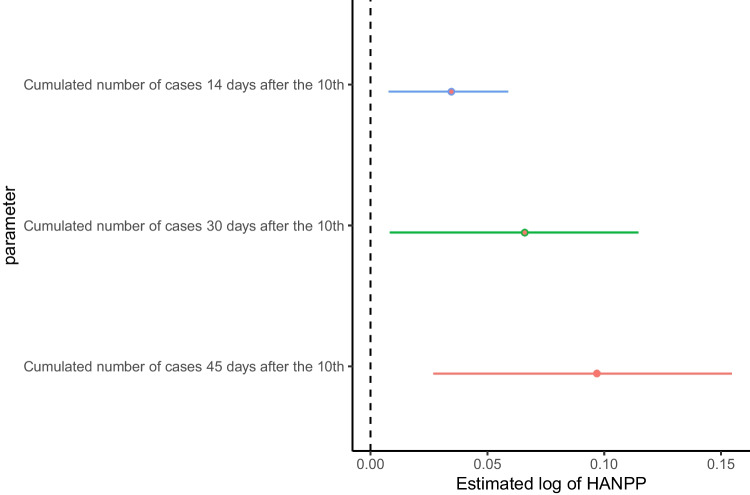
Estimated coefficients of the log of HANPP (mean and 95% confidence intervals). Note: Results are based on estimates that include country random effects

We then come to the focus of our paper, i.e. the hypothesis that human overexploitation of natural resources being an accelerating factor of Covid-19 diffusion, by estimating the coefficient of HANPP. The coefficient estimate is positive and significant in all the specifications, supporting our research question about the potential role of human impact on the ecosystem in Covid-19 early diffusion. This result provides evidence of a possible nexus between ecosystem degradation due to human activities and the propagation of the pandemic at the (global) regional level, which further confirms the results obtained by Fernández et al. (2021) at (global) country level. For example, in correspondence of model (ii) characterized by the lowest DIC, we find that a 1% increase of HANPP (expressed in Gtc/yr) is associated with a 0.035% increase in Covid-19 early diffusion. However, looking at Fig. 3, in which we report the estimated coefficient of the log of HANPP for the cumulative cases after 10, 30 and 45 days from the detection of the first ten cases, we observe an increase in its impact.^15^ Indeed, after 30 days, a 1% increase of HANPP is associated with a 0.066% increase in Covid-19 early diffusion, thus almost doubling the previous result, and further increasing to threefold 0.097% after 45 days.

Finally, the reliability of this result is reinforced by the design of our analysis, which is based on a worldwide regional sample that accounts for 730 regions in 63 countries, and offers a large sample size to compute accurate estimates once controlling for heterogeneity by the inclusion of random effects.

## Conclusion

In this paper we study the effect of ecosystem degradation on the spread of Covid-19 virus. We provide evidence of this link assessing the effect of human overexploitation of natural resources, measured by the Human Appropriation of Net Primary Production (HANPP) index, on the number of Covid-19 cumulative cases registered in the following 14 days from the date when > 10 cumulative cases were reported for 730 regions in 63 countries till May 30, 2020. Our result, robust against a wide range of control variables and several alternative estimation techniques, is part of a larger body of scientific literature supporting the connection between over-exploitation of land and the spread of zoonoses.

From a policy perspective, our contribution emphasises that the debate should move towards a more robust understanding of human impacts on ecosystems and health consequences, adopting an interdisciplinary approach to land-use planning, agriculture and environmental protection, while ensuring food security and public health. The need to feed a growing world population and ensure sustainable global economic development must be reframed within a framework that respects the natural evolution of ecosystems and protects biodiversity. It is therefore necessary to rethink our production and consumption patterns, both globally and individually, to make them more prudent in their use of water and other natural resources, but also to encourage the reduction of food and waste in general. Responsible land management must be placed at the heart of future policy agendas now, otherwise the fundamental role of ecosystem services in providing resilient solutions and natural barriers to current, and likely future, zoonotic emergence will be seriously threatened (Everard et al., 2020). In this regard, governments around the world, through the scale and pace of actions they have taken in response to the Covid-19 pandemic, have demonstrated that they have the institutional capacity to provide substantive and coordinated responses to external global threats, whenever they are perceived as such. This shows that it is crucial to improve the perception of the importance of environmental issues. Demonstrating the close link between environmental degradation and Covid-19, a traumatic health event with profound social and economic consequences, goes precisely in this direction.

Finally, given the cross-sectional nature of our study, although the time lag of the HANPP helps in the direction of interpreting our results as causal, we cannot explicitly claim for it. To achieve this aim we would require data with a time dimension to identify the dynamics associated with the phenomena, as well as different econometric methodologies capable of isolating the impact of a specific covariate once the full set of relevant exogenous determinants is taken into account. Our study, therefore, could be conceived as a first step in demonstrating the relationship between environmental degradation and the Covid-19 pandemic, which will need to be further explored. The next steps could be to move beyond a causal approach and consider the complex circular relationship between ecosystem resilience and air pollution, including a taxonomy of the latter. Another possibility, probably very promising, would be to consider ecosystems as mediators of the effect of pollution on the spread of Covid-19.

## Data Availability

Data are freely available at the following links: Https://globaldatalab.org/ Https://github.com/ec-jrc/ COVID-19 Https://sedac.ciesin.columbia.edu/data/collection/hanpp Http://cidportal.jrc.ec.europa.eu/ftp/jrc-opendata/GHSL/GHS_POP_GPW4_GLOBE_R2015A/GHS_POP_GPW42015_GLOBE_R2015A_54009_1k/V1-0/GHS_POP_GPW42015_GLOBE_R2015A_54009_1k_v1_0.zip\

## Appendix A

Table 3

**Table 3:** Correlation matrix

	(a)	(b)	(c)	(d)	(e)	(f)	(g)	(h)	(i)	(j)	(k)	(l)	(m)	(n)
(a) Cumulative Cases	1.0000													
Demographic Variables														
(b) log Total Population	0.1621	1.0000												
(c) Population Age 65 +	0.0341	-0.1845	1.0000											
(d) Proportion Males	0.0559	0.1554	-0.3483	1.0000										
Socio-Economic Variables														
(e) Prevalence Diabetes	0.0168	0.3143	-0.414	0.2591	1.0000									
(f) log Number of Test	-0.0052	-0.1388	0.1298	-0.4306	-0.1331	1.0000								
(g) Stringency Index	-0.0212	-0.1733	-0.3289	-0.069	-0.0161	0.1421	1.0000							
(h) log Income Index	0.0978	-0.1586	0.5311	-0.1296	-0.172	0.2661	-0.3847	1.0000						
(i) log Schooling Level	0.0081	-0.2962	0.5626	-0.3846	-0.2806	0.3746	-0.2872	0.7534	1.0000					
(j) log Pop. Wtd. Density	0.0421	0.4368	-0.4865	0.2053	0.1541	-0.2249	0.0647	-0.481	-0.5618	1.0000				
Environmental Variables														
(k) Temperature	-0.1086	0.0176	-0.4275	0.2512	0.3304	-0.3574	0.0978	-0.4892	-0.4897	0.2831	1.0000			
(l) Humidity	0.0506	-0.0534	-0.155	0.3744	-0.1127	-0.2411	-0.0383	-0.0603	-0.2116	0.1149	0.2319	1.0000		
(m) PM 2.5 Concentration	0.1729	0.6042	-0.2445	0.3471	0.2714	-0.1333	-0.0609	-0.2074	-0.3632	0.4569	0.0159	0.002	1.0000	
(n) log HANPP	0.1254	0.4083	0.1797	0.0761	0.0495	-0.3222	-0.3501	0.2181	0.0545	0.1889	0.1207	0.0841	0.3183	1.0000

## Appendix B: Robustness check

In this section we present the outcome of the robustness checks executed to reinforce the reliability of our results. More in detail, we have re-estimated twice the six model specifications detailed in Sect. 3, using as the dependent variable the cumulative number of cases 30 and 45 days after the 10th case is observed. Results of these robustness checks are shown in Tables 4 and 5 respectively.

Table 4

**Table 4:** Estimation results considering the cumulated number of cases 30 days after the 10th

Model Specification	Baseline			Country R.E			Continent + Nested Country R.E		
	(i)			(ii)			(iii)		
Variable	**Mean**	**0.025 q**	**0.975 q**	**Mean**	**0.025 q**	**0.975 q**	**Mean**	**0.025 q**	**0.975 q**
Intercept	**-20.2047**	**-28.7000**	**-11.6625**	**-10.5377**	**-18.8072**	**-2.2079**	**-20.1990**	**-28.9000**	**-11.4874**
Demographic Variables									
log Total Population	**0.6865**	**0.5810**	**0.7913**	**0.5556**	**0.4372**	**0.6726**	**0.6864**	**0.5790**	**0.7933**
Population Age 65 +	**0.0328**	**0.0002**	**0.0659**	-0.0158	-0.0677	0.0359	0.0328	-0.0005	0.0666
Proportion Males	0.0376	-0.1000	0.1760	0.0441	-0.0759	0.1633	0.0376	-0.1030	0.1787
Socio-Economic Variables									
Prevalence Diabetes	0.0304	-0.0246	0.0855	-0.0991	-0.2416	0.0390	0.0304	-0.0257	0.0866
log Number of Test	0.0050	-0.0242	0.0334	-0.0087	-0.0458	0.0281	0.0050	-0.0248	0.0339
Stringency Index	**0.0181**	**0.0097**	**0.0263**	-0.0075	-0.0207	0.0052	**0.0181**	**0.0095**	**0.0264**
log Income Index	**1.3519**	**1.0200**	**1.6753**	0.2860	-0.1335	0.7039	**1.3519**	**1.0200**	**1.6817**
log Schooling Level	0.0074	-0.0696	0.0852	**0.2235**	**0.1025**	**0.3445**	0.0075	-0.0710	0.0868
log Population Weighted Density	**0.1508**	**0.0147**	**0.2902**	-0.0273	-0.1681	0.1144	**0.1509**	**0.0121**	**0.2930**
Environmental Variables									
Temperature	0.0088	-0.0127	0.0300	0.0165	-0.0058	0.0386	0.0089	-0.0131	0.0304
Humidity	**0.0139**	**0.0020**	**0.0255**	**0.0127**	**0.0012**	**0.0241**	**0.0139**	**0.0018**	**0.0257**
PM 2.5 Concentration	-0.0028	-0.0072	0.0018	**0.0074**	**0.0028**	**0.0121**	-0.0028	-0.0073	0.0019
log HANPP	-0.0794	-0.1680	0.0037	**0.0660**	**0.0083**	**0.1146**	-0.0794	-0.1700	0.0053
Country random effects				0.9795	0.5288	1.6255	0.9796	0.5287	1.6254
DIC	8100.1			7720.8			7720.8		

Model Specification	Spatial R.E			Spatial R.E Country F.E			Spatial R.E. + Continent F.E. + Country F.E		
	(iv)			(v)			(vi)		
Variable	**Mean**	**0.025 q**	**0.975 q**	**Mean**	**0.025 q**	**0.975 q**	**Mean**	**0.025 q**	**0.975 q**
Intercept	**-20.1990**	**-28.9000**	**-11.4874**	-6.6490	-27.1347	13.8203	-6.8314	-42.9200	29.2277
Demographic Variables									
log Total Population	**0.6864**	**0.5790**	**0.7933**	**0.5304**	**0.4028**	**0.6566**	**0.5304**	**0.4028**	**0.6566**
Population Age 65 +	0.0328	-0.0005	0.0666	-0.0294	-0.0932	0.0342	-0.0295	-0.0933	0.0341
Proportion Males	0.0376	-0.1030	0.1787	0.0219	-0.1057	0.1487	0.0219	-0.1057	0.1486
Socio-Economic Variables									
Prevalence Diabetes	0.0304	-0.0257	0.0866	-0.0685	-3.2034	3.0638	-0.0284	-3.5672	3.5074
log Number of Test	0.0050	-0.0248	0.0339	-0.0040	-0.0440	0.0360	-0.0040	-0.0440	0.0360
Stringency Index	**0.0181**	**0.0095**	**0.0264**	**-0.0172**	**-0.0339**	**-0.0012**	**-0.0172**	**-0.0339**	**-0.0012**
log Income Index	**1.3519**	**1.0200**	**1.6817**	0.0058	-0.4525	0.4662	0.0054	-0.4527	0.4657
log Schooling Level	0.0075	-0.0710	0.0868	**0.2468**	**0.1114**	**0.3820**	**0.2468**	**0.1114**	**0.3819**
log Population Weighted Density	**0.1509**	**0.0121**	**0.2930**	-0.0028	-0.1534	0.1482	-0.0027	-0.1533	0.1483
Environmental Variables									
Temperature	0.0089	-0.0131	0.0304	**0.0259**	**0.0019**	**0.0496**	**0.0259**	**0.0019**	0.0496
Humidity	**0.0139**	**0.0018**	**0.0257**	0.0098	-0.0024	0.0218	0.0098	-0.0024	0.0218
PM 2.5 Concentration	-0.0028	-0.0073	0.0019	**0.0087**	**0.0036**	**0.0138**	**0.0087**	**0.0036**	**0.0138**
log HANPP	-0.0794	-0.1700	0.0053	**0.0757**	**0.0169**	**0.1250**	**0.0757**	**0.0169**	**0.1250**
Country random effects									
DIC	8098.9			7721.2			7721.1		

bold indicates coefficients statistically different from zero at 5% level.

Table 5

**Table 5:** Estimation results considering the cumulated number of cases 45 days after the 10th

Model Specification	Baseline			Country R.E			Continent + Nested Country R.E		
	(i)			(ii)			(iii)		
Variable	**Mean**	**0.025 q**	**0.975 q**	**Mean**	**0.025 q**	**0.975 q**	**Mean**	**0.025 q**	**0.975 q**
Intercept	**-25.4333**	**-36.2584**	**-14.6016**	**-14.5877**	**-24.0861**	**-5.0457**	**-14.5923**	**-24.0882**	**-5.0528**
Demographic Variables									
log Total Population	**0.8532**	**0.7129**	**0.9900**	**0.5975**	**0.4654**	**0.7278**	**0.5975**	**0.4655**	**0.7279**
Population Age 65 +	0.0271	-0.0202	0.0747	-0.0166	-0.0749	0.0415	-0.0166	-0.0749	0.0414
Proportion Males	0.1718	-0.0035	0.3472	0.1029	-0.0334	0.2382	0.1029	-0.0333	0.2382
Socio-Economic Variables									
Prevalence Diabetes	0.0187	-0.0458	0.0839	-0.0705	-0.2453	0.1036	-0.0705	-0.2448	0.1032
log Number of Test	0.0188	-0.0198	0.0560	-0.0106	-0.0523	0.0311	-0.0106	-0.0523	0.0311
Stringency Index	**0.0176**	**0.0064**	**0.0287**	-0.0134	-0.0278	0.0005	-0.0134	-0.0277	0.0005
log Income Index	**1.0155**	**0.6121**	**1.4142**	0.3701	-0.1158	0.8580	0.3705	-0.1151	0.8581
log Schooling Level	0.0383	-0.0647	0.1413	**0.2450**	**0.1022**	**0.3882**	**0.2449**	**0.1021**	**0.3880**
log Population Weighted Density	**0.1949**	**0.0326**	**0.3618**	-0.0207	-0.1726	0.1327	-0.0207	-0.1726	0.1327
Environmental Variables									
Temperature	0.0046	-0.0220	0.0309	**0.0368**	**0.0111**	**0.0622**	**0.0368**	**0.0111**	**0.0622**
Humidity	-0.0013	-0.0172	0.0143	0.0020	-0.0124	0.0160	0.0020	-0.0124	0.0160
PM 2.5 Concentration	**-0.0092**	**-0.0158**	**-0.0023**	0.0018	-0.0045	0.0084	0.0018	-0.0045	0.0084
log HANPP	-0.0338	-0.1480	0.0718	**0.0969**	**0.0269**	**0.1547**	**0.0968**	**0.0269**	**0.1547**
Country random effects				0.7066	0.3822	1.1729	0.7063	0.3821	1.1717
DIC	6606.8			6248.6			6248.5		

Model Specification	Spatial R.E			Spatial R.E. + Country F.E			Spatial R.E. + Continent F.E. + Country F.E		
	(iv)			(v)			(vi)		
Variable	**Mean**	**0.025 q**	**0.975 q**	**Mean**	**0.025 q**	**0.975 q**	**Mean**	**0.025 q**	**0.975 q**
Intercept	**-25.4553**	**-35.9447**	**-14.9638**	-13.6000	-36.2478	9.1218	-13.6000	-54.3485	27.1818
Demographic Variables									
log Total Population	**0.8533**	**0.7175**	**0.9860**	**0.5690**	**0.4289**	**0.7078**	**0.5690**	**0.4290**	**0.7078**
Population Age 65 +	0.0271	-0.0187	0.0732	-0.0169	-0.0869	0.0522	-0.0170	-0.0870	0.0521
Proportion Males	**0.1721**	**0.0023**	**0.3420**	0.0925	-0.0524	0.2365	0.0924	-0.0525	0.2364
Socio-Economic Variables									
Prevalence Diabetes	0.0187	-0.0438	0.0818	0.0394	-3.4043	3.4802	0.0425	-3.9810	4.0625
log Number of Test	0.0188	-0.0186	0.0549	-0.0163	-0.0608	0.0283	-0.0163	-0.0608	0.0283
Stringency Index	**0.0176**	**0.0068**	**0.0284**	**-0.0174**	**-0.0337**	**-0.0017**	**-0.0174**	**-0.0337**	**-0.0017**
log Income Index	**1.0148**	**0.6240**	**1.4010**	0.1810	-0.3602	0.7279	0.1800	-0.3604	0.7275
log Schooling Level	0.0383	-0.0615	0.1380	**0.2970**	**0.1366**	**0.4570**	**0.2970**	**0.1366**	**0.4570**
log Population Weighted Density	**0.1948**	**0.0375**	**0.3563**	-0.0027	-0.1661	0.1620	-0.0025	-0.1660	0.1621
Environmental Variables									
Temperature	0.0046	-0.0212	0.0301	**0.0450**	**0.0172**	**0.0725**	**0.0450**	**0.0172**	**0.0725**
Humidity	-0.0013	-0.0167	0.0138	0.0001	-0.0151	0.0150	0.0001	-0.0151	0.0150
PM 2.5 Concentration	**-0.0092**	**-0.0157**	**-0.0026**	0.0015	-0.0058	0.0089	0.0015	-0.0058	0.0089
log HANPP	-0.0337	-0.1442	0.0688	**0.1060**	**0.0340**	**0.1645**	**0.1060**	**0.0340**	**0.1645**
Country random effects									
DIC	6605.4			6251.8			6251.8		

bold indicates coefficients statistically different from zero at 5% level

## Footnotes

1 See, among others, the discussion provided by Kremen (2015) on land-sparing and land-sharing for biodiversity conservation.

2 An overview for the EU is provided by Staniszewski et al. (2023) who assess structural conditions over the time period 2005–2018. Beltran-Peña et al. (2020) study the effects of agriculture on food self-sufficiency in the twenty-first century in 165 countries designing different future scenarios considering changes in diet habits, population, agricultural intensification, and climate, highlighting criticalities on the side of crop production, food imports, and arising of food supply shocks. Thomson et al. (2019), in turn, stress “the importance of a careful assessment of environmental impacts of emerging technology and management across scales to achieve sustainable intensification of land” in the future, while Basso and Antle (2020) focus on digital agriculture in the design of sustainable agricultural systems.

3 Citing Woolhouse et al. (2012), Dobson et al. (2020) also report that “for a century, two new viruses per year have spilled from their natural hosts into humans”, with the risk of virus spread increasing as human-wildlife interactions become more frequent.

4 Following Burkhard et al. (2012): “ecosystem services are the contributions of ecosystem structure and function – in combination with other inputs – to human well-being.” See also Bennett et al. (2015) for a further detailed definition and analysis, and Baudron and Liégeois (2020) for additional evidence on land use change and other drivers of zoonotic infection diseases.

5 The Projecting Responses of Ecological Diversity in Changing Terrestrial Systems (PREDICT) database is a geographically and taxonomically representative database for spatial comparisons of biodiversity. It has been created to support research in ecology and conservation biology for the understanding of the global status of biodiversity (Hudson et al., 2017).

6 “NPP is the net amount of carbon assimilated in a given period by vegetation. It determines the amount of energy available for transfer from plants to other levels in the trophic webs in ecosystems.” (Haberl et al., 2007). Detailed definitions are also provided by Díaz (2003) and Sun et al. (2022).

7 Specifically, the HANPP accounts for the grams of carbon per grid cell produced by vegetal food, fibre and grain fed to livestock. For wood, fuel wood and paper products, organic matter was added to account for processing and harvest losses. For paper, recycling was also considered.

8 For example, Lombardy region, which lies in the Po Valley in Italy and concentrates the highest share of population, industries and intensive livestock farms in the country, scores among the regions with the highest value of HANPP by Imhoff et al. (2004) and with the lowest value of HANPP by Kastner et al. (2021).

9 For example, Shahzad et al. (2020) find a mixed effect of average temperature on COVID-19 incidence across Chinese provinces, while Yao et al. (2020) find no significant association across a sample of Chinese cities. On the other hand, a positive association between temperature and COVID-19 transmission is found in Japan (Azuma et al., 2020), Pakistan (Raza et al., 2021) and India (Singh et al., 2021).

10 Other air pollutants such as ozone (O3), carbon monoxide (COx) and nitrogen monoxide (NH3) may also lead to higher numbers of confirmed COVID-19 cases and fatalities (for a review, see Han et al., 2022).

11 Following Besag et al. (1991), the spatial random effects for region *i* conditional to region *j* follow a Gaussian distribution as follows:

$${u}_{i}|{u}_{j},{\tau }_{u}\sim N\left(\frac{1}{\sum_{j=1}^{m}{\upomega }_{ij}}\sum\nolimits_{j\epsilon {\delta }_{i}}{\upomega }_{ij}{u}_{j},\frac{1}{{\tau }_{u},{n}_{{\delta }_{i}}}\right) i\ne j$$

where, *τ*_*u*_ is the conditional precision of spatial random effects and *δ*_*i*_ is the neighbourhood of the *i*^th^ region, *n*_*δi*_ is the number of neighbours, $\mathrm{\omega}_{ij}$ is an element of the spatial weight matrix **W** where region *j* is considered a neighbour of region *i* if it is among the 5 nearest neighbours.

12 In our model the degree of overdispersion of our count data is assumed to be constant across regions. This limitation is due to the cross-sectional characteristic of the data. Other authors like Rui et al. (2021) take advantage of the time dimension of the data by analysing the spread of Covid-19 in the US via a spatio-temporal multivariate time series model accounting for heterogeneous overdispersed count data. In our framework, we account for unobserved heterogeneity by taking into account extra spatial variability in the data and testing different ways of modelling spatial random effects.

13 Corona et al. (2021) in a systematic review and meta-analysis of 3714 articles show that diabetes is the most important cause of mortality in COVID-19 hospitalised patients in both the US and Europe.

14 Although people or workers’ mobility across municipalities or regions has been verified to be an important determinant of the risk of disease diffusion, at least at national or local level (see one for all, Ascani et al., 2021), it is not observable for much of our sample, and we exclude this variable from the set of exogenous factors. However, population density and the stringency index allow to pick up at least part of the phenomenon, given that the latter is measuring a restriction in mobility during the first wave.

15 Results are based on estimates that include country random effects. The whole set of results is in Table 4 and Table 5 in Appendix B.

## Declarations

All authors have read, understood, and have complied as applicable with the statement on "Ethical responsibilities of Authors" as found in the Instructions for Authors and are aware that with minor exceptions, no changes can be made to authorship once the paper is submitted.
